# Predicting deep-brain stimulation frequencies to suppress pathological population oscillations in a network model of Parkinson's disease

**DOI:** 10.1186/1471-2202-14-S1-P291

**Published:** 2013-07-08

**Authors:** Abbey B Holt, Theoden I Netoff

**Affiliations:** 1 Department of Neuroscience, University of Minnesota, Minneapolis, Minnesota, 55455, USA; 2 Department of Biomedical Engineering, University of Minnesota, Minneapolis, Minnesota, 55455, USA

## 

Deep-brain stimulation (DBS) is used to treat medication-refractory Parkinson's disease (PD). However, tuning stimulation parameters requires time intensive visits with a clinician using a trial-and-error process until therapy is achieved with minimal side effects [1]. There is a need for a systematic approach to determining DBS parameters from the patient's physiological response to the stimulus. It is hypothesized that oscillations in the basal ganglia are responsible for many motor signs of PD [2] and that DBS works by disrupting these pathological oscillations. These oscillations arise from the excitatory-inhibitory loop between subthalamic nucleus (STN) and globus pallidus external (GPe) [3]. Periodic forcing of an oscillating system can induce chaos at certain frequencies [4]. When the system is chaotic it behaves aperiodically thereby suppressing the pathological oscillations. From a system's phase response curve (PRC), it is possible to determine at which stimulus frequencies and amplitudes chaotic responses will occur. Here we measure PRCs and predict optimal stimulation frequencies in a physiologically realistic computational model of STN DBS [5]. The model consists of 300 neurons in GPe and 100 neurons in STN and was tuned to reproduce non-human primate data. In the PD state, GPe activity produces a strong 32 Hz oscillation similar to the beta oscillations observed in the animal model. DBS pulses are applied to the STN and its efferent connections. Stimulation at 136 Hz, commonly used clinically, suppresses the beta oscillation. From low frequency stimulation (2 Hz) we are able to estimate the PRC of the population oscillation to the DBS pulse. From the measured PRC the stimulus frequencies that induce chaos in the STN-GPe loop are predicted. The STN was then stimulated over a range of frequencies and the power of the 32 Hz population oscillation measured. There is a strong correlation between the stimulus frequencies predicted to produce chaos from the PRC and the simulations where beta is suppressed. This indicates that it may be possible to predict DBS frequencies to successfully eliminate pathological oscillations based on a PRC measured from a patient using low frequency stimulation. Novel contributions of this work are methods of measuring PRCs from population activity and utilizing them to accurately predict stimulus frequencies that suppress oscillations in a model of the basal ganglia. We propose that this approach can be applied to patients in which electrophysiology measurements can be made simultaneously with stimulation. This approach to tuning stimulation parameters may dramatically reduce tuning time and improve efficacy for treatment of PD.
